# Cluster randomized trial of influenza vaccination in patients with acute heart failure in China: A mixed-methods feasibility study

**DOI:** 10.1371/journal.pgph.0001947

**Published:** 2023-06-16

**Authors:** Rong Liu, Xin Du, Anushka Patel, Gian Luca Di Tanna, Yangyang Zhao, Zhiyan Wang, Yihang Fan, Hao Zhang, Yang Yi, Jianzeng Dong, Craig Anderson, Hueiming Liu

**Affiliations:** 1 Heart Health Research Center (HHRC), Beijing, China; 2 The George Institute for Global Health, UNSW, Sydney, Australia; 3 Beijing Anzhen Hospital, Beijing, China; 4 Department of Innovative Technologies, University of Applied Sciences and Arts of Southern Switzerland (SUPSI), Lugano, Switzerland; 5 The University of California, Irvine, California, United States of America; 6 The University of Pittsburgh, Pittsburgh, Pennsylvania, United States of America; 7 The First Affiliated Hospital of Zhengzhou University, Zhengzhou, Henan Province, China; 8 Menzies Centre for Health Policy and Economics, University of Sydney, Sydney, Australia; Sciensano, BELGIUM

## Abstract

Uncertainties about the efficacy of influenza vaccination for populations with heart failure (HF) in preventing cardiovascular outcomes, as well as lack of effective vaccination strategies, may contribute to low vaccine coverage rate (VCR) in China and globally. We assessed the feasibility of a strategy to promote influenza vaccines in patients hospitalized with acute HF in China and to inform the design of a hybrid effectiveness-implementation cluster randomized trial to evaluate this strategy on mortality and hospital re-admission. We conducted a cluster randomized pilot trial involving 11 hospitals in Henan Province in China, with mixed-methods evaluation between December 2020 and April 2021. A process evaluation involved interviews with 51 key informants (patients, health professionals, policy makers). The intervention included education about influenza vaccination and availability of free vaccines administered prior to hospital discharge for HF patients, while usual care included attending community-based points of vaccination (PoV) for screening and vaccination. Implementation outcomes focused on reach, fidelity, adoption, and acceptability. Recruitment rates were assessed for trial feasibility. Effectiveness outcomes were influenza VCR, HF-specific rehospitalizations and mortality at 90 days. A total of 518 HF patients were recruited from 7 intervention and 4 usual care hospitals (mean of 45 participants per hospital per month). VCR was 89.9% (311/346, 86.1–92.8%) in the intervention group and 0.6% (1/172, 0.0–3.7%) in the control group. The process evaluation demonstrated reach to patients with lower socioeconomic and education status. There was good fidelity of the intervention components, with education and PoV set up processes being adapted to local hospital workflow and workforce capacity. Intervention was acceptable and adopted by patients and health professionals. However, outside of a trial setting, concerns were raised around vaccination reimbursement costs, workforce accountability and capacity. The intervention strategy appears feasible and acceptable for improving VCR in HF patients at county-level hospitals in China.

**Trial registration:** This pilot trial is registered with the acronym PANDA II Pilot (Population Assessment of Influenza and Disease Activity) at ChiCTR.org.cn (ChiCTR2000039081).

## Introduction

Global guideline recommendations for the use of annual influenza vaccination in the elderly and among high-risk patients is particularly pertinent to patients with heart failure (HF), where disease progression is often exacerbated by respiratory infections [1–3]. However, influenza vaccine coverage rates (VCR) in people with HF vary widely across the globe, [4] and are particularly low (~0.6%) in China. Potential reasons include low awareness of influenza vaccination, uncertainty about benefits, limited access to vaccines, and relatively high out-of-pocket costs (80 to 200 Chinese Yuan [USD 13–31]) [4–6]. Influenza vaccination is available for people at Point of Vaccination (PoV) located in Centers for Disease Control and Prevention (CDCs) or in community health centers in China [7]. Influenza vaccination is only free of charge in several cities in China, and rarely in smaller administrative jurisdictions such as counties, for older adults (age ≥ 60 years); [8] making vaccination access a barrier since county hospitals cater to 75% of the Chinese population [9, 10].

Given there is uncertainty about the efficacy of influenza vaccination in preventing mortality and readmission among HF patients, and the effectiveness of strategies to improve influenza vaccine coverage in this patient population in China, policy- and practice-relevant evidence are required. Prior to initiating a large-scale hybrid effectiveness-implementation trial, we sought to assess the feasibility of a complex intervention aiming to influence patient, provider and system level behaviors. Specifically, in a multicenter pilot study, we aimed to 1) to assess recruitment feasibility; 2) estimate parameters to inform the sample size calculation for a large-scale trial; and 3) to understand the feasibility, acceptability, barriers and facilitators of this complex intervention to inform any modifications prior to the large-scale trial.

## Methods

### Ethics statement

The pilot trial was approved by Medical Ethics Committee of Beijing Anzhen Hospital Affiliated to Capital Medical University (approval number of ks2020017) and the Human Genetics Resources Administrative Office of the Chinese Ministry of Science and Technology (approval number 2020, GH3553). The process evaluation was approved by Medical Ethics Committee of Beijing Anzhen Hospital Affiliated to Capital Medical University (approval number 2020096X) and by Human Research Ethics Committee at the University of New South Wales (approval number HC200988).

### Design

We did a convergent mixed methods study that included a pilot cluster randomized trial and an embedded process evaluation [11, 12] The two-arm, parallel, hospital-based, pilot cluster randomized trial compared an intervention package for improving influenza vaccine uptake to current usual care. County-level hospitals were eligible if they treated an average of >35 HF patients per month during the planned recruitment months in previous years, had clinical staff available to be involved, were able to adhere to the study protocol, and were likely to be able to follow-up participants in the community. Eligible participants were patients hospitalized with HF severity defined by New York Heart Association (NYHA) classification grades III-IV at the time of admission between December 2020 and January 2021. Patients with a known allergy to vaccine components or who were pregnant were excluded. Patients were followed up at 1-month and 3-months post-discharge. The process evaluation was a qualitative study involving key informant interviews with relevant stakeholders and was informed by the UK Medical Research Council (MRC) process evaluation framework for complex interventions [13].

### Randomization

The unit of randomization was the hospital. Eleven hospitals were assigned in a ratio of 2:1 by computer-generated random numbers (7 intervention and 4 control). More sites were assigned to the intervention arm to maximise variation of contexts in which intervention implementation could be observed.

### Recruitment

Consecutive patients diagnosed with heart failure by their treating physicians were screened against the study eligibility criteria. The diagnosis of HF was based on typical symptoms and signs according to the 2016 European Society of Cardiology HF guidelines [14]. This clinical diagnosis was validated in each case by a trained cardiologist based on record review and clinical judgement. A prior history of hypertension, diabetes, atrial fibrillation, or stroke was captured by patient self-report, based on knowledge of these conditions, year of diagnosis and relevant treatments. While rehospitalizations were captured during follow-up, recurrent hospitalizations of the same patient due to HF during the recruitment period were not included.

### Intervention

**Fig 1:**
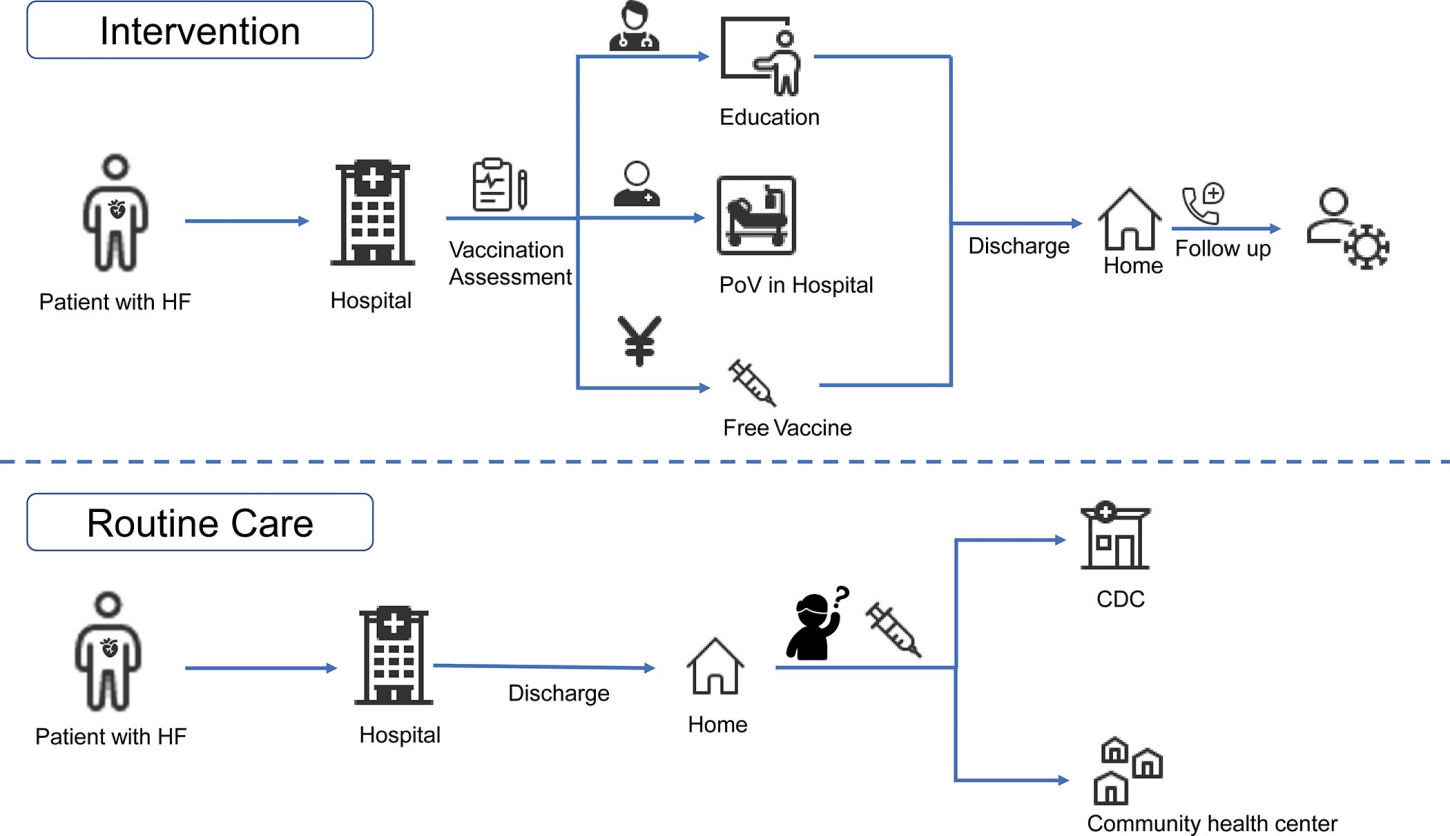
Comparison of patient journeys in the intervention strategy and routine care.

The intervention package (**Fig 1 and S1 Text**) included (i) education of the health care team and patients; (ii) establishment of an immunization service within the hospital; and (iii) provision of free influenza vaccine on the day of hospital discharge. Hospitals randomized to the control group continued usual care, which generally would be advice at discharge to attend a community based PoV service, where they would have incurred out-of-pocket expenditure for the influenza vaccine.

### Outcomes

Implementation outcomes focused on reach, fidelity, adoption, and acceptability. Recruitment rates were assessed for trial feasibility. The primary quantitative outcome was influenza vaccine coverage. Secondary outcomes were influenza like illness (ILI) and the composite of death or HF-specific hospital readmission for heart failure at 3-months follow up. ILI was defined as an acute respiratory illness with a measured temperature of ≥ 38°C and cough, at any time during hospitalization [15]. Survival status at discharge and at 3-months follow-up was collected, with details of post-discharge events obtained through face-to-face or telephone interviews with the patient or next-of-kin. Data was collected on recruitment rate (number of participants recruited per month), and loss to follow-up at a hospital level.

Vaccination status was recorded by site investigator and checked against immunization history registry and receipt from health facilities providing vaccination service. Clinical outcomes reported by patients during follow up were verified by site investigator based on electronic medical records.

### Statistical analysis

Descriptive analysis included VCR (percentage), recruitment rate (consented participants per month), ILI rate (percentage), and 3-month death or HF readmission rate (percentage). To assess the associations between a series of outcomes and the randomized group we have fitted the following: logistic regression for influenza vaccination status, Poisson model for ILI and death or HF-specific hospital readmission and Proportional-Hazards Cox regression with shared frailty for time to death or HF-specific readmission. All models were hierarchical at patient level with hospital as random-effects to take account of the cluster effect. Covariates included in the models were age, sex, education level, insurance type, history of coronary heart disease, history of hypertension, history of diabetes, ejection fraction category, brain natriuretic peptide level, and pharmaceutical treatment. Binary outcomes are reported as proportions with 95% confidence intervals (CI) calculated using the Wilson Score method with continuity correction. Intra-class correlation (ICC) for the outcome of death or HF-specific readmission at 3-months post-discharge was estimated to inform the sample size calculation for the planned subsequent large-scale trial. All statistical analyses were undertaken using R 3.6.1 (R Foundation for Statistical Computing, Vienna, Austria, https://www.R-project.org/).

### Process evaluation

The process evaluation was informed by the UK MRC process evaluation guidance [13] framework which supports a systematic examination of the relationships between contextual factors, intervention, implementation fidelity, intervention’s mechanism of impact, and outcome. [13] The consolidated criteria for reporting qualitative research (COREQ) was used in reporting the process evaluation [16].

Interview guides were developed by three primary researchers RL, HL, and AP, based on the process evaluation framework and included questions about health system context, acceptability of the intervention and barriers and facilitators to its implementation (see **S2 and S3 Texts**). The interview guides were piloted with two participants, with edits made to enhance clarity.

Maximum purposive sampling was used across key characteristics which included intervention and control sites, how well the intervention was implemented (for intervention sites), whether the site was in a county location that was above or below gross domestic product (GDP) per capita (as a proxy for the socioeconomic characteristic of the geographic area), and type of stakeholder (patients and health professionals). Both vaccinated and unvaccinated patients were sampled.

Health professionals were recruited with the assistance of site principal investigators and other investigators. All invited participants agreed to an interview and provided informed consent.

Team reflexivity: RL and ZW have backgrounds in public health and clinical medicine, and conducted the interviews. Patient and health professional participants across all centers were recruited by YZ, a clinical research coordinator who knew some but not all the participants prior to the interviews, and introduced RL and ZW to interviewees before interview.

Face to face interviews for health professionals took place at interviewees’ workplaces. Patients were invited to a hospital office and a family member was often present during interviews involving older patients. Interviews were conducted either video or telephone call for participants who could not attend in person. All interviews were audio recorded except for one interviewee who only agreed to a written record of the interview. Interviews, each of which lasted about 30 minutes, were conducted from March 8th, 2021 to April 22nd, 2021.

### Analysis of the qualitative data

Deductive coding was applied by the team to the interview transcripts to generate a set of key themes based on the MRC framework, followed by inductive coding to add new codes [17]. Initially, three interviews (a healthcare provider, a public health professional, and a patient) were independently coded by RL, YF, HZ, YY. The team discussed their codes and generated a coding framework for the remaining interviews. Transcription and analyses were done in Chinese, and the coding framework was translated to English. Selected illustrative quotes were translated to English and back translated to Chinese to check for accuracy by bilingual researchers. Description of the coding tree is provided in the Supporting Information. Nvivo 12 (QSR International Pty Ltd. 2020) was used for data management and analysis.

Themes from the qualitative study were triangulated with the quantitative pilot trial data to understand fidelity of the intervention components, and the mechanisms of the acceptability and adoption of the intervention contributing to the trial outcomes [18].

### Reporting guidelines

This study reporting follows the standard protocol items of the Recommendations for Interventional Trials (SPIRIT), Consolidated Standards of Reporting Trials (CONSORT) statements, and Template for Intervention description and Replication (TIDieR) [19–21]. This pilot trial is registered at ChiCTR.org.cn (ChiCTR2000039081) and the acronym is PANDA II Pilot (Population Assessment of Influenza and Disease Activity).

## Results

### Participant characteristics and VCR

**Fig 2:**
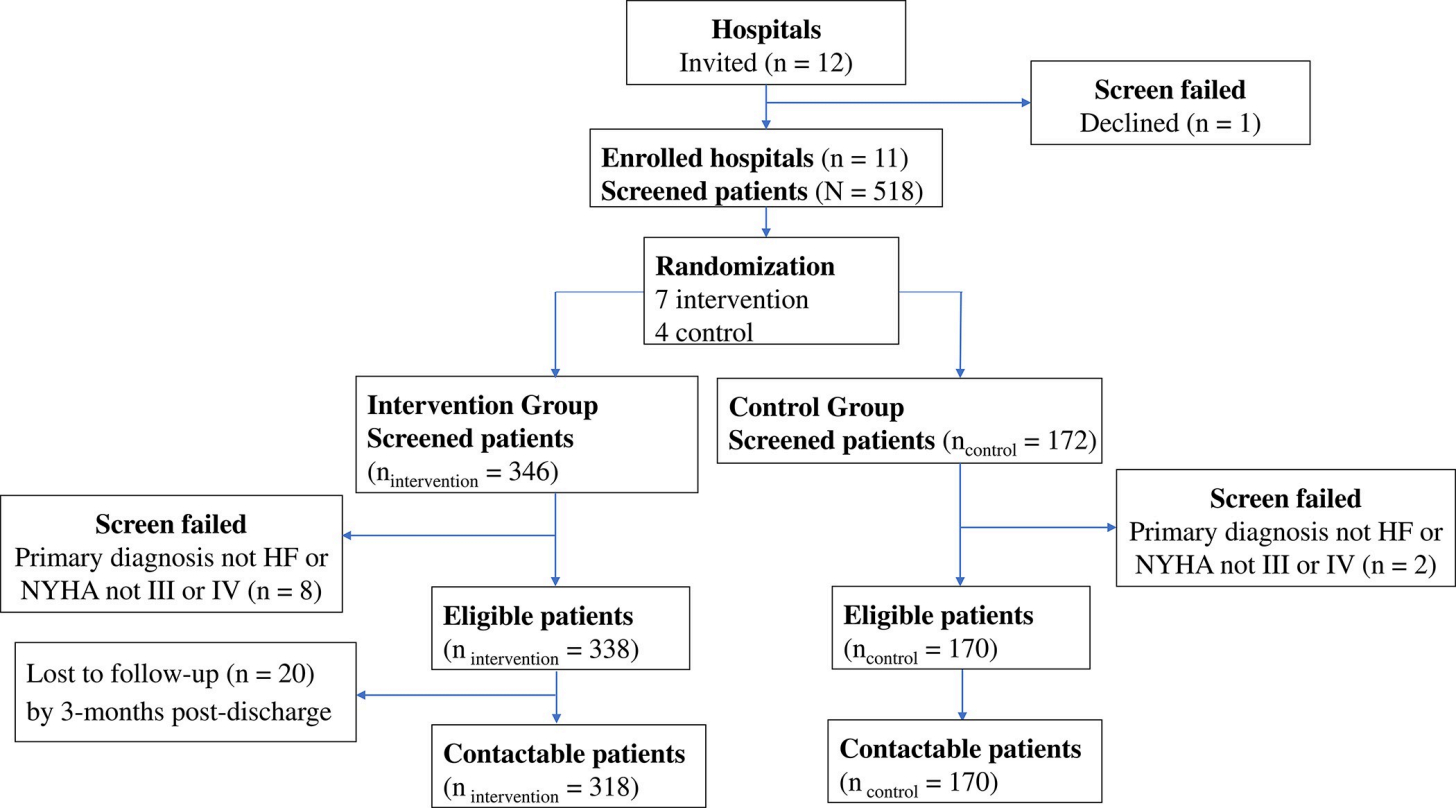
Patient flowchart. (Analyses were done on 518 patients’ full dataset with intention to treat principle).

A total of 518 patients with hospitalized HF were recruited from 11 hospitals in Henan Province, China between December 28^th^, 2020 and January 29^th^, 2021 (mean 1.4 patients per hospital per day, range 0.6–1.8). A mean of 47 patients (range 22–61) were recruited per hospital. **(Fig 2). Table 1** summarizes the characteristics of participants: median age of 72.8 years, 52.7% were male, 71% had a primary school or lower level of education, and 82.6% were covered by a rural cooperative medical insurance plan. Overall, the median length of hospitalization was 9 days.

**Table 1:** Baseline patient characteristics for participants in PANDA II pilot study.

Variables	Intervention	Control
**Age, years (median [IQR])**	73.5 (66.8, 80.0)	73.9 (63.8, 79.4)
**Female, n (%)**	158 (46.7%)	81 (48.5%)
**Disease history**		
** Coronary artery disease, n (%)**	106 (31.1%)	46 (27.2%)
** Myocardial infarction, n (%)**	61 (17.9%)	18 (10.7%)
** Percutaneous coronary intervention, n (%)**	30 (8.8%)	18 (10.7%)
** Coronary artery bypass graft, n (%)**	10 (2.9%)	2 (1.2%)
** Other coronary disease, n (%)**	85 (24.9%)	34 (20.1%)
** Hypertension, n (%)**	147 (43.1%)	92 (54.4%)
** Diabetes mellitus, n (%)**	71 (20.8%)	39 (23.1%)
** Hyperlipidemia, n (%)**	10 (2.9%)	8 (4.7%)
** Atrial fibrillation, n (%)**	94 (27.6%)	44 (26%)
** Congenital heart disease, n (%)**	8 (2.3%)	4 (2.4%)
** Cerebrovascular disease, n (%)**	46 (13.5%)	32 (18.9%)
** Respiratory disease, n (%)**	57 (16.8%)	46 (27.2%)
** Mental illness, n (%)**	0 (0%)	2 (1.2%)
**Ejection fraction, % (median [IQR])**	40 (39, 59)	46 (37, 57)
** Ejection fraction < 40%, n (%)**	96 (29.8%)	49 (32.9%)
**Elevated BNP or NT-proBNP, n (%)**	235 (69.7%)	112 (67.1%)
**Length of stay, days (median [IQR])**	8 (7, 11)	9 (7, 12)
**Use antibiotics, n (%)**	138 (41.3%)	71 (42.5%)
** Fever, n (%)**	13 (3.8%)	6 (4.3%)
** Elevated white blood cell, n (%)**	59 (17.5%)	28 (16.8%)
**In-hospital death, n (%)**	6 (1.8%)	3 (2.2%)
**Prescribed medication at discharge**		
** Beta blocker, n (%)**	209 (62.8%)	97 (75.2%)
** ACE inhibitor/ ARB/ ARNI, n (%)**	151 (45.5%)	92 (71.9%)
** ACE inhibitor, n (%)**	49 (14.7%)	20 (15.9%)
** ARB, n (%)**	48 (14.5%)	32 (25.4%)
** ARNI, n (%)**	54 (16.3%)	40 (31.7%)
** MRA, n (%)**	389 (80.8%)	120 (93.0%)
** Diuretics, n (%)**	231 (69.4%)	127 (96.2%)

Data are presented in median (inter-quartile range limits) or count (percentage).

IQR, Inter-quartile range; BNP, Brain-type Natriuretic Peptide; NT-proBNP, N-terminal pro-B-type Natriuretic Peptide; ACE, Angiotensin-Converting Enzyme; ARB, Angiotensin II Receptor Blocker; ARNI, Angiotensin Receptor/Neprilysin Inhibitor; MRA, Mineralocorticoid Receptor Antagonist.

All hospitals in the intervention arm were able to establish local PoVs, even within cardiology wards, which included obtaining a permit from CDC, management of influenza vaccine stock and cold chain, and training existing nurses to immunize, over a two-to-four-week period.

### Outcomes

VCR in the intervention group was 89.9% (311/346), compared to 0.6% (1/172) in control sites.

**Table 2** provides details of clinical outcomes. At 3-month post-discharge, follow-up data for 498 patients (96.1%) were available.

**Table 2:** Outcomes for participants in PANDA II pilot study.

Outcomes	Intervention	ICC	Control	ICC
**Influenza vaccination, n (%)**	311/346, (89.9%)	0.06 (0, 0.52)	1/172, (0.1%)	-
**Influenza-like illness, n (%)**	13/346, (3.8%)	-	6/172, (3.5%)	-
**Death or HF readmission, n (%)**	87/346, (25.1%)	0.10 (0, 0.40)	44/172, (25.6%)	-

ICC, Intra-class correlation coefficient. ICCs were not estimable for outcomes in some groups.

During hospitalization, 19 patients (3.7%) reported having an ILI. At 3-month follow-up, death or HF readmission occurred in 25.3% (131/518) participants, which includes 7.1% of participants who died and 19.1% of participants who were re-hospitalized. There were no significant differences in any clinical outcome between randomized groups (**S1 Table**).

#### Findings from the process evaluation

**Fig 3:**
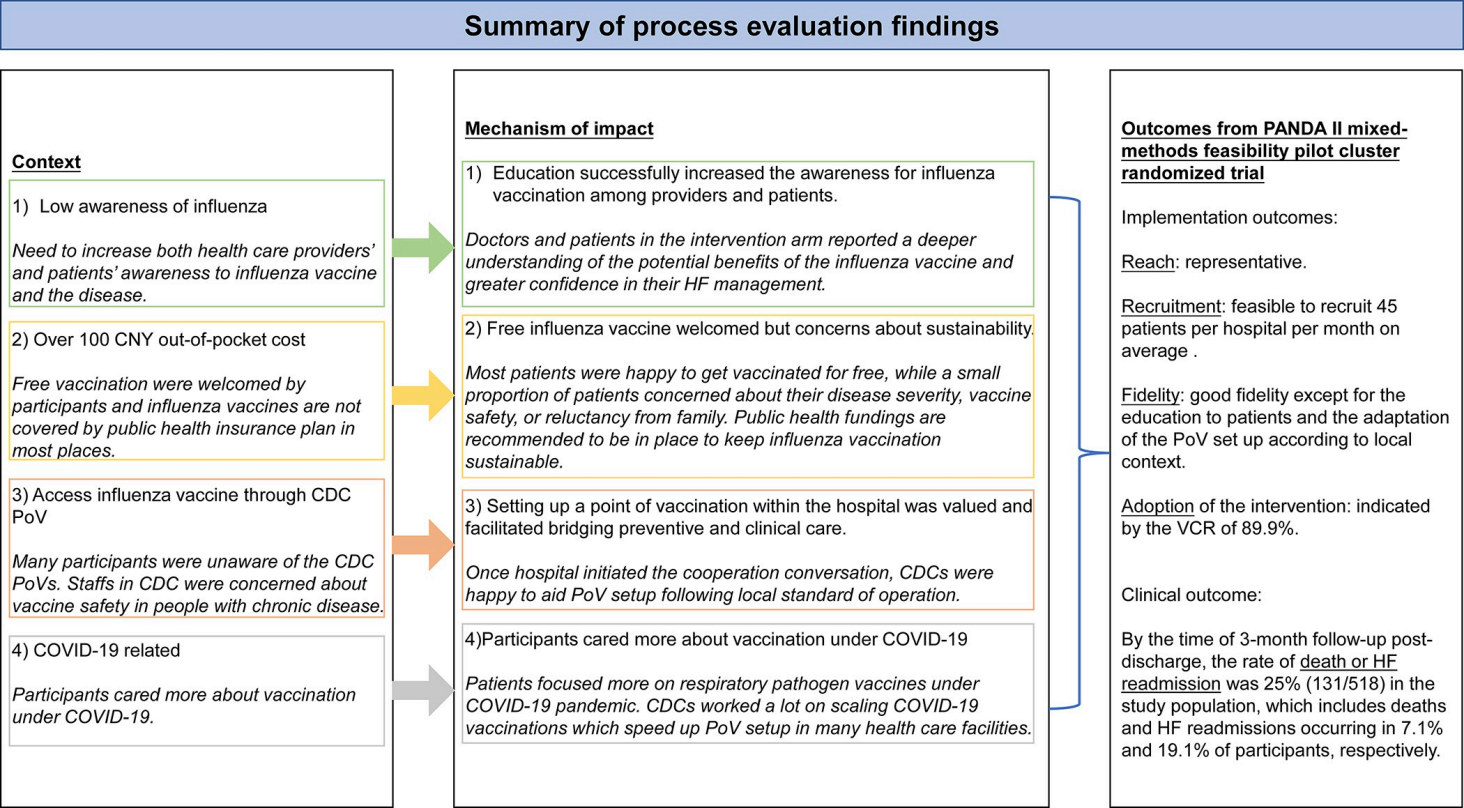
Summary of process evaluation findings (in italics) as compared against our hypothesized contextual assumptions and causal mechanism (in non-italics) in impacting upon the outcomes of PANDA II pilot cluster randomized trial.

Participants involved in the interviews included patients (n = 13), doctors (n = 6), nurses (n = 5), hospital managers (n = 13), public health professionals (n = 9), health department and public health insurance bureau staffs (n = 5) across 9 sites, representing sites from low, middle, and high economic development areas, with poor and good compliance, from both arms (**S2 Table**). The triangulation of the themes across context, mechanisms of the acceptability and adoption, resulting in the outcomes of the pilot trial are summarized in **Fig 3.** Additional illustrative quotes across the themes are included in **S3 Table**.

#### Reach and recruitment

Baseline characteristics of pilot participants are broadly similar to those of participants from a recent large prospective HF registry designed to represent patients within the same province of China [22], except for lower education level and greater rural health insurance plan coverage in the current study, which purposefully focused exclusively on county-level hospitals. It was also noted that recruitment was challenging due to the COVID-19 pandemic, with less patients presenting to hospital due to higher admission criteria than usual for less urgent patients, longer admission screening time including COVID-19-related tests, and doctors being seconded as surge staff to provide COVID-19-related services outside cardiology wards. Nevertheless, it was still feasible to recruit enough patients for pilot study.

#### Fidelity and local adaptation of the intervention components

*Education*. Educational workshops were delivered to both control and intervention sites according to protocol. However, there was limited use of the patient educational materials on influenza vaccines and heart failure, as these were not distributed to the patients, by most of the site investigators. Instead, to streamline it with their clinical work, they verbally informed patients about importance of influenza vaccination upon admission, during the consent process, and at the time of discharge.

*Point of vaccination to be set up within the hospital*. The vaccination of participants was undertaken in different formats, with adaptation to local health system context: in-house public health unit delivery of vaccines at the bedside in cardiology wards, cardiology nurses vaccinating patients at the bedside, and patients being transferred to PoV in another location in the hospital.

*Provision of free vaccination*. Free influenza vaccination at discharge was offered to all eligible participants, and was administered to 89.9% of participants in the intervention arm. About 10% of participants in the intervention arm refused to be vaccinated mainly due to perceived uncertain interactions between vaccination and scheduled interventional medical treatments in addition to pharmacological therapeutics, concerns over vaccine safety, family advice, or no perceived benefit.

#### Mechanisms affecting the acceptability and adoption of intervention components

*Education successfully increased the awareness of the benefits for influenza vaccination among providers and patients*. Education regarding influenza vaccine, cardiovascular disease outcome, HF risk factors, and the value of disease prevention, were provided to patients and health care providers in both intervention and control arms. Routine care in county level hospitals rarely includes education on influenza vaccination for HF patients, as discharge plans mainly comprised of information on HF triggers such as respiratory infection, excessive salt intake, and other noncardiac causes, but did not cover the importance of influenza vaccination. Only a few patient interviewees were aware of the association between influenza and cardiac health, or about how and where to access influenza vaccination, before the study intervention. One patient from the intervention site said, *‘I think it is useless to get influenza vaccine*. *I don’t always get cold during winter*, *so it is the same for me no matter I get influenza vaccination or not*.*’* In comparison, doctors and patients in the intervention arm reported a deeper understanding of the potential benefits of the influenza vaccine after prescribing it to the patients or receiving it themselves and greater confidence in their HF management. A doctor from an intervention hospital said, *‘Being a clinical doctor and treating patients every day*, *I sometimes found myself lacking the knowledge of disease prevention and control*. *I had no idea that there were vaccines until I heard about it from CDC staffs and education workshops*.*’*

However, health professionals from CDC offices expressed concern about the potential for adverse events following the immunization of patients with chronic disease, and were unsure about the optimal timing of the influenza vaccine and wanted more advice from patients’ treating physicians. Similarly, the immunization nurses described being uncomfortable in determining the eligibility of the heart failure patients for influenza vaccines, and wanted greater guidance from the treating clinicians.

Both patients and health professionals highlighted how the quality of the doctor-patient therapeutic relationship was key as patients who trusted their doctors were more likely to agree to the influenza vaccine, and that including family and extended family members was important in the patient’s decision making. Few hospitals incorporate routine follow-up clinic visits to manage HF patients, which were incorporated within the intervention strategy as a means to collect clinical outcomes and further turned out to be acceptable by strengthening doctor-patient therapeutic relationship.

*Free influenza vaccine welcomed but concerns about sustainability*. Health professionals, patients, and policy makers generally welcomed the opportunity for free influenza vaccination. However, outside a trial setting, policy makers described that subsidizing free influenza vaccination of patients with chronic diseases was not a priority for the allocation of limited resources, as compared to pharmaceutical treatment, because of perceived greater importance of treatment over prevention and uncertain efficacy of influenza vaccines. Many health providers and patients indicated the need for the cost of vaccinations, to be kept low in areas with limited resources, to overcome initial hesitancy towards vaccine due to out of pocket costs, and to enable the county hospitals to feasibly incorporate influenza vaccination into their routine care. Health commission staff described how the economic development status of the county affected the ability of public insurance schemes to fund vaccination programs. It was noted that more affluent patients are more likely to self-purchase influenza vaccines each year to prevent exacerbation of chronic diseases. Health commission staff recommended the need for public medical insurance to cover vaccines to improve awareness and further vaccine coverage in patients and carers.

A health commission staff from a control county said, *‘For most people*, *a hundred RMB is a large cost*. *They have a lot to bear*. *If those in authority could provide financial assistance or medical insurance support*, *the work would be easily achieved*.*’*

*Setting up a point of vaccination within the hospital was valued and facilitated bridging preventive and clinical care*. CDC officers and hospital managers valued the convenience of vaccinating patients around the time of hospital discharge, and perceived that PoVs have the potential to benefit other at-risk chronic disease patient groups beyond HF patients after acute treatment. Hospital leadership championed the establishment of an in-hospital PoV service and associated resources. Most health providers described increasing health literacy as a high priority, particularly in rural areas where there is poor adherence to disease management plans and delayed presentation for acute events in the context of low education and knowledge. This study set an example for them to plan and utilize preventive care services in clinical settings, as a hospital manager from intervention site put *‘as a result of national fundamental policies*, *(the hospital) now prioritize prevention and combines prevention with treatment*. *This (current pilot study) is a great project that we can easily embrace*.*’* However, providers highlighted that adding preventive care to the heavy workload of clinicians might not be sustainable beyond the pilot study. Some doctors expressed concerns over accountability of the vaccination and suggested assigning specialized staff if it was to become routine care.

A doctor from an intervention hospital reinforced how the project facilitated inter-department collaboration. *‘Our cardiology department chair and the head of the hospital immunization service department consult with each other in terms of vaccination for patients*.*’*

### Influenza vaccination in the context of the COVID-19 pandemic

In the context of the COVID-19 pandemic, some of the PoVs setup were leveraging off the existing COVID-19 vaccination spots. Temporary PoVs were set up to respond COVID-19 pandemic, either in a public health unit or in immunization tent in the open areas in hospitals (such as gardens or small parks between hospital buildings). These temporary PoVs were already certified by local CDC for COVID-19 vaccination, therefore administratively adding the influenza vaccination to the existing COVID-19 vaccination certification was easier than starting from scratch setting up a brand new PoV.

A nurse from an intervention hospital said, *‘Then*, *through this program*, *explain to the patient*. *He/she (patient) would understand after the explanation that the vaccine could not be taken whenever you wished (supply constraints and during flu season)*. *The patient showed that he/she was aware of the situation and that he/she was pleased to be vaccinated*.*’*.

## Discussion

The pilot study showed that timely recruitment of participants for a well-powered clinical outcomes trial is feasible. The implementation of the trial intervention is largely feasible with some adaptations relating to establishing a PoV. Using data from this pilot study, we propose a large-scale hybrid efficacy-effectiveness cluster randomized trial involving 24 ~158 hospitals in each arm and a mean of 50 patients per hospital to detect a 7% ~ 18% relative risk reduction of 1-year mortality and readmission in intervention group participants compared to those in control group over three influenza seasons (trial registration number: ChiCTR2100053264). The main trial will contribute to a growing body of efficacy data in diverse populations, as well as contribute data on the effectiveness of an implementation strategy in a specific setting, in addition to recent efficacy findings of preventing cardiovascular outcomes from meta-analysis of RCTs and observational study [23, 24]. Moreover, the main trial will provide more information on vaccine safety and cost-effectiveness for intervention scaleup.

Recruitment was sufficiently timely despite the COVID-19 pandemic, which reflects the substantial prevalence and readmission rate of heart failure in China. In 2017, the prevalence of heart failure in China was estimated to be 1.1% and 1-year readmission rate around 50% [25]. The total population of the pilot trial counties is at least 300,000, which may equate to 3,000 prevalent HF patients [9]. Assuming one-third of these patients require hospitalization at any given time, admission rates of at least two per day may be expected. The observed recruitment rate of 1.4 patients per day seen in this study is consistent with such estimates.

These data provide some reassurance given the challenges experienced in recruitment in other recent influenza vaccination trials. The multinational IAMI trial included a sample size of 2571 of the prespecified 4400 participants recruited between October 2016 to March 2020, with COVID-19 pandemic impacts during the latter period of recruitment [26, 27] The IVVE trial, a multinational trial which included a tertiary hospital in Beijing China, reached a sample size of 5129 participants with HF including 694 from China, comparing to the prespecified target of 5000 participants [28, 29]. Despite the encouraging pilot data, recruitment for our planned large-scale trial may still be impacted by infectious disease mitigation measures, because low level of respiratory virus circulation, will make it hard to detect any effects on clinical outcomes prevented by influenza vaccination with less or no influenza viruses circulating around [30].

There are few prior randomized controlled trials evaluating a complex intervention to improve influenza vaccine coverage in cardiovascular patients. The intervention in this study aimed to address three barriers to influenza vaccination, namely low awareness, lack of access and unaffordability. Previous studies in various settings showed less VCR changes using different interventions. In 2014/2015 influenza season, a pre-post quasi-experimental study of an intervention of healthcare worker recommendation and temporary PoV, improved VCR to 19% post-intervention, compared to 0.3% pre-intervention, among community-dwelling older adults in China [6]. While the COVID pandemic may have had some impact on VCR in usual care, this is unlikely to have been a key factor given other data indicating similar coverage in Chinese populations before the pandemic and as recently as 2017 [4, 31]. A cluster randomized trial in New Zealand showed that free influenza vaccines improved the VCR to 45% compared to 17% in usual practice in a general practitioner setting [32]. A national-level policy change evaluation in Europe, including free influenza vaccine, showed VCR decreased in the majority of countries after seven years since 2007/2008 influenza season, which potentially could be due to an ineffective vaccination campaign, though no clear reasons were presented from the report [33].

The adaptation of the Intervention components by the health providers, indicates that a level of adaptation of the core components, tailored to the local site context would be required in the hybrid effectiveness-implementation trial. Engaging potential end users to refine an intervention showed feasibility to improve VCR to 74% in children and older adults, compared to 37% VCR in standard of care group, in primary care setting at urban Guangzhou China, in 2020/2021 influenza season [34]. The same study conducted at rural setting for its pilot phase showed a higher VCR of 91%, similar as our pilot study, in its intervention group. Non-traditional settings for influenza vaccination provision, including pharmacies, the home, street corners, have been shown to be potentially feasible among underserved population, but these have not been evaluated in controlled studies [35, 36].

This pilot study has several limitations. The overlapping involvement of staff in both the intervention delivery and study procedure may limit “real world” applicability and therefore its effectiveness. For example, staff took the opportunity to deliver education on influenza vaccination at the time of obtaining informed consent for study participation. Educating the patients as part of the informed consent may need to be incorporated into normal nursing care in a real-world setting. The feasibility shown in a small number of pilot hospitals might not be generalizable to other settings in China, although they were selected to reflect some diversity.

While this pilot trial suggests feasibility and acceptability of the vaccination strategy in hospitals, if the large-scale trial demonstrates effectiveness in preventing clinical events, sustainable scale-up will need to consider and address broader issues highlighted in the process evaluation. These include the need for policies and public funding to subsidize vaccinations, efforts towards health system integration, and wider medical education and health promotion to improve professional and community understanding of the risks and benefits of influenza vaccination, particularly amongst high-risk individuals.

## Conclusion

Based on this pilot trial, an adequately powered hybrid effectiveness-implementation trial of influenza vaccination in patients with heart failure can be conducted in county hospitals in China. The process evaluation suggests that the proposed intervention strategy to ensure uptake of influenza vaccination in patients with heart failure may also be feasible. However, careful consideration about equitable funding sources for influenza vaccination, service integration, health promotion is needed for future sustainable scaleup.

## Supporting information

S1 Checklist CONSORT 2010 checklist. (DOC)

S1 File A protocol of a cluster randomized pilot trial evaluating the feasibility of intervening heart failure patients with influenza vaccines (Population Assessment of iNfluenza and Disease Activities, PANDA II pilot). (PDF)

S1 Data Data of PANDA II pilot study. (XLSX)

S1 Text Intervention details. (DOCX)

S2 Text Interview guide for health professionals. (DOCX)

S3 Text Interview guide for patients. (DOCX)

S1 Table Regression models output summary for outcomes. (DOCX)

S2 Table Interviewees characteristics. (DOCX)

S3 Table Additional illustrative quotes from interviewees. (DOCX)
