# Supplementary material for: Cluster randomized trial of influenza vaccination in patients with acute heart failure in China: A mixed-methods feasibility study
Source: PLOS Glob Public Health. 2023 Jun 16;3(6):e0001947. doi: 10.1371/journal.pgph.0001947 (PMC10275428; doi:10.1371/journal.pgph.0001947)
Supplement: S1 File — (PDF) [file pgph.0001947.s002.pdf]

A protocol of a cluster randomized pilot trial evaluating the feasibility of intervening heart failure patients with influenza vaccines (Population Assessment of iNfluenza and Disease Activities, PANDA II pilot)

Roles and Responsibilities

Jianzeng Dong, The First Affiliated Hospital of Zhengzhou University, Principal Investigator;
Xin Du, Heart Health Research Center (HHRC), Principal Investigator;
Craig Anderson, Heart Health Research Center (HHRC), Co-Principal Investigator; Professor of Neurology and Epidemiology, Faculty of Medicine, UNSW Sydney; Neurologist, Royal Prince Alfred Hospital, Sydney Health Partners; Honorary Professor, Sydney Medical School, University of Sydney; Executive Director, George Institute China at Peking University Health Science Center.
C. Raina Macintyre, Head, Biosecurity Research Program, Kirby Institute, UNSW Medicine.
Gian Luca Di Tanna, Head of Statistics & Associate Professor, The George Institute for Global Health, Faculty of Medicine, University of New South Wales, Australia.

The study sponsor, Heart Health Research Center (HHRC), is responsible for the study design, committee coordination, data and project management, assistance with the ethics committee and the Administration of Human Genetic Resources applications, trial procedure development, site training, data and safety monitoring, adherence to protocol, data analysis, preparing manuscript, and making decision to submit for publication.

Data Safety Monitoring Board (DSMB)

Yi (Jake) Sui, Director, Shenyang Brain Institute; Chief Director, Department of Neurology and Neuroscience, Shenyang First People's Hospital; Shenyang Brain Hospital; Shenyang Medical College Affiliated Brain Hospital.
Bruce Neal, Executive Director, George Institute Australia; Professor of Medicine, UNSW Sydney; Honorary Professor, Sydney Medical School, University of Sydney; Professor of Clinical Epidemiology, Imperial College London.
John Chalmers, Professor of Medicine, UNSW Sydney; Emeritus Professor of Medicine, University of Sydney.
Xian Li, Head of Statistics, The George Institute for Global Health (China)

Glossary of abbreviations and terms

Abbreviation	Definition
ACEI	Angiotensin Converting Enzyme Inhibitor
AEFI	Adverse Event Following Immunization
AMR	Antimicrobial Resistance
ARNI	Angiotensin Receptor Neprilysin Inhibitor
CDC	Center for Disease Control and Prevention
CI	Confidence Interval
CONSORT	The Consolidated Standards of Reporting Trials
CHF	Congestive Heart Failure
CRF	Case Report Form
DSMB	Data and Safety Monitoring Board
EDC	Electronic Data Capture
ERC	Ethic Review Committee
GWTG-HF	Get With The Guidelines – Heart Failure
HF	Heart Failure
HGRAC	Human Genetic Resources Administration of China
HHRC	Heart Health Research Center
HR	Hazard Ratio
ILI	Influenza-like Illness
IRB	Institutional Review Board
NYHA	New York Heart Association
OR	Odds Ratio
PARADIGM-HF	Prospective Comparison of ARNI with ACEI to Determine Impact on Global Mortality and Morbidity in Heart Failure
POV	Point of Vaccination
SPIRIT	Standard Protocol Items: Recommendations for Interventional Trials
VCR	Vaccine Coverage Rate

Protocol Synopsis

**Title**	**(Population Assessment of iNfluenza and Disease Activities, PANDA II pilot)**
**Clinical phase**	IV
**Rationale**	Considerable data support the broad benefits of influenza vaccination in the general population. However, there is uncertainty over the effectiveness of influenza vaccination in preventing cardiovascular events in the high-risk population such as those with HF. Influenza vaccination is not covered by medical insurance in majority of China. The rate of influenza vaccination was as low as 0.6% in Chinese patients with HF, compared to about 60% in the Western European countries and in the US. Thus, it is of critical importance to detect whether providing free influenza vaccine, providing vaccination service in hospital, and educating patients as well as health provider will increase vaccine rate among patients hospitalized due to severe symptoms of HF. If so, whether improved influenza vaccine rates in patients with HF will reduced all-cause death or HF hospitalization. Before a definitive randomized cluster randomized trial, a pilot study is planned to address the following uncertainties for the design of future definitive study:<br>(i) Whether the intervention package is feasible;<br>(ii) Whether a target influenza vaccine rate of 90% is achievable in the intervention clusters;<br>(iii) Proportion of patients hospitalized with HF getting influenza vaccination in routine care;<br>(iv) Number of patients will be enrolled in future study.
**Study design**	Two-arm, parallel, hospital-based, cluster randomized trial.
**Intervention**	(i) education of the health care team and patients; (ii) provision of free vaccine on the day of discharge; and (iii) provision of immunization service inside hospital before discharge.
**Number of participants**	330 patients; recruitment from 11 hospitals in one influenza season (average of 30 patients per hospital).
**Study duration**	12 months
**Endpoints/Outcomes**	*Primary outcomes*: influenza vaccine coverage rate (VCR) in both groups.<br>*Secondary outcomes*: number of patients recruited during study period, rate of patient follow-up, death or readmission for HF over 3 months follow-up.

**Site inclusion criteria**	• Capable of admitting, treating, recruiting HF patients;<br>• With the capacity of treating >35 HF patients per month during recruitment period;<br>• Staff with available care team personnel to collaborate and control the lost to follow-up rate to <10%.<br>• Local approval to provide influenza vaccination from administrative bureau.
**Site exclusion criteria**	• Participating in vaccination-related or other HF patient outcome improvement programs that might confound study outcomes.
**Patient inclusion criteria**	• All adult inpatients (age ≥18 years) discharged with a diagnosis of HF during recruitment month in participating hospital;<br>• Assessed as New York Heart Association (NYHA) III-IV during hospitalization;<br>• Consent to participate in the study.
**Patient exclusion criteria**	• Known allergy to influenza vaccine;<br>• Pregnant women.
**Intervention administration**	Staff of the participating hospitals.
**Safety evaluation**	Adverse Event Following Immunization (AEFI) will be monitored and reported.
**Study schedule**	Pilot phase December 1, 2020 - November 30, 2021.<br>Each patient will be followed for 3 months.
**Statistical considerations**	Rates of influenza vaccination (the number of patients received influenza vaccine divided by the number of patients enrolled in the intervention group and in the control group, will be reported respectively.<br>Number of patients enrolled in both the intervention and the control group will be reported.<br>Rate of patient follow-up at 3 months after enrollment will be reported.
**Data Management**	A web-based electronic data capture system will be used to store and check data.

Background

Heart failure (HF) is a major clinical and public health concern. An estimated 1.3% (13.7 million) of the adult population (age ≥35 years) have HF in China. [1] Burden of hospitalizations and health care costs among individuals with HF in China are higher than those in other low and middle income countries. [2] About one-third of HF associated hospitalizations are triggered by respiratory infection. [3,4] Considerable data support the broad benefits of influenza vaccination in the general population. [5] However, there is uncertainty over the efficacy of influenza vaccination for preventing cardiovascular events in the high-risk patients with HF. [6,7] Although Guidelines for HF management and guidelines for other cardiovascular disease management recommend influenza vaccine for patients with cardiovascular disease, there are scare of evidence-based data as to the effect of influenza vaccination in patients with HF.

Associations between influenza and cardiovascular disease varied from study to study. In a retrospective cohort, for patients with existing congestive heart failure (CHF), the overall hospitalization rate during influenza season was 11% higher (Hazard Ratio [HR] 1.11, 95% confidence interval [CI] 1.03-1.20) than that during non-influenza season. [8] A population-level estimate of influenza-associated excess cardiovascular hospitalization accounted for 10% (95% CI, 7%-14%) in the oldest old. [9] However, all-cause hospitalization wasn't found to be associated with influenza vaccination in HF patients in a post hoc analysis of the Prospective Comparison of ARNI with ACEI to Determine Impact on Global Mortality and Morbidity in Heart Failure (PARADIGM-HF) study. This secondary analysis from trial data showed influenza vaccination was associated with lower risk of all-cause death (HR 0.82, 95% CI 0.70-0.96), but not with lower risk of cardiovascular death or HF-related hospitalization, or all-cause hospitalization. [10] A nationwide cohort study suggested similar 18% reduced risk of all-cause death in HF patients receiving ≥ 1 influenza vaccination (HR 0.82; 95% CI, 0.81–0.84). [7] HF inpatients with concomitant influenza infection were reported to have higher in-hospital mortality than those without concomitant influenza infection (6.2% vs. 5.4%, odds ratio [OR] 1.15, 95% CI 1.03 to 1.30). [11] However, a data linkage study from the Get With The Guidelines – Heart Failure (GWTG-HF) quality improvement initiative showed similar 1-year all-cause mortality and 1-year all-cause readmission between influenza vaccinated HF inpatients and those unvaccinated. [12] Given the above inconsistent results observed in different studies, definitive conclusions cannot be drawn without high quality randomized clinical trials.

Influenza vaccination is more commonly reimbursed for both the elderly and those with chronic conditions by public funding in the Americas and the Western Europe than in the Western Pacific. [13,14] A government run insurance scheme for influenza vaccines seems correlates more with a higher influenza vaccine coverage rate, than with economic development status across countries. [15] In China, the free influenza vaccine policy for people older than 60 years old is only applied in a few cities. [16] The rate of influenza vaccination was as low as 0.6% in Chinese patients with HF, compared to about 60% in the Western European countries and in the US.[10] Given the WHO recommendation that all people except those younger than 6 months should get influenza vaccine, the low influenza vaccine rate in China provides us a chance to study the efficacy of influenza

vaccination in reducing mortality and HF hospitalization, as neglectable low proportion of HF patients received the influenza vaccine in the routine care.

Under current vaccine regulations in China, adult immunization service can only be sought at licensed community health center or immunization clinics housed in center for disease control and prevention (CDC). Hospitals are allowed to establish a temporary Point of Vaccination (POV) after a quality check and then approval valid for three months from their local department of health. But most hospitals don't have a POV in place at the time as many of them consider vaccination being out of the scope of clinical services. Unaware of the importance of annual vaccinations, not knowing where to get vaccinated, and not wanting to pay are the top barriers prohibiting those with chronic conditions from getting influenza vaccines. [17] Thus, it is of critical importance to investigate whether improved influenza vaccine coverage rate in patients with HF will reduced all-cause death or HF hospitalization. Before investigating the efficacy of influenza vaccines on preventing cardiovascular disease, we need to first address whether educating patients and health provider, providing vaccination service in hospital, and providing free influenza vaccine will achieve a target vaccine coverage rate (VCR) among patients hospitalized due to severe symptoms of HF.

Before a definitive cluster randomized trial, a pilot feasibility study is planned to address the following uncertainties: 1) Whether the intervention package is feasible; 2) Whether a target influenza vaccine rate of 90% is achievable in the intervention clusters; 3) Proportion of patients hospitalized with HF getting influenza vaccination in routine care; 4) Number of patients will be enrolled in future study.

Methods

This protocol follows the extension to CONSORT 2010 statement on reporting pilot and feasibility trials, the SPIRIT 2013 statement on standard protocol items for clinical trials, and its explanation and elaboration report. [19-21]

Trial design

This pilot trial is a two-arm, parallel, hospital-based, cluster randomized trial where 7 hospitals will be randomized to intervention and 4 hospitals to control arm. All eligible HF inpatients from each hospital will be recruited during 2020 November. Intervention includes (i) education of the health care team and patients; (ii) provision of free vaccine on the day of discharge; and (iii) provision of immunization service inside hospital before discharge.

Setting

PANDA II pilot will be conducted in county-level hospitals in Henan Province, China. County-level hospitals serve a relatively fixed population living in the corresponding geographic area, which to the largest extent prevents recruiting the same patients seeking care in different participating centers.

<u>Inclusion criteria for participating sites</u>:
1) capable of admitting and treating HF patients;

2) with the capacity of treating >35 HF patients per month during recruitment period;
3) staff with available care team personnel to collaborate.
4) local approval to provide influenza vaccination from administrative bureau/ center for disease control and prevention (CDC).

Exclusion criteria for participating sites:
Participation in a vaccination-related HF patient outcome improvement programs that might confound study outcomes.

Patients
PANDA II pilot trial will focus on HF patients at higher risk of having serious complications from influenza infection. HF is largely a clinical diagnosis. Based on the widely used Boston criteria, HF will be defined according to the attending physician's report of any of the following clinical symptoms described as dyspnea ('unusual' on light exertion or recurrent in the supine position); fluid retention; description of 'rales' in the lungs, jugular venous distension, or pulmonary edema on physical examination; or pulmonary edema on chest x-ray presumed related to cardiac dysfunction. [22] HF patients will be identified through screening the admission list of a cardiovascular ward in each participating hospital for the following eligibility criteria.

Inclusion criteria for participating patients:
1) all the adult inpatients (age ≥18 years) with a discharge diagnosis of HF during recruitment month in participating hospitals;
2) assessed as New York Heart Association (NYHA) function classification III-IV during hospitalization;
3) consent to participate in the study.

Exclusion criteria for participating patients:
1) have known allergy to influenza vaccine.
2) pregnant women.

Intervention
The intervention will include three components:

1) education to all members of the health care team, physicians, nurses, and patients;
    a. A 30-minute training session to health care teams in all participating hospitals after they are included in the study. Members in a health care team include site investigators, physicians, and nurses. The training session include the association between influenza vaccine and lower risk of mortality and rehospitalization in patients with HF, the study protocol, and current regulations on vaccine circulates. The education material will be designed and delivered by study investigators.
    b. Study nurses in each participating hospital will lead a 15-minute group or individual training session to patients after they reach a relative-stable status during hospitalization. The training sessions include plain language version content on association between influenza infection and

cardiovascular disease and current guideline recommendations of influenza vaccine for secondary prevention in patients with established cardiovascular disease. The training material (presentation slides, scripts, and videos) will be designed and delivered by study investigator.

2) provision of free influenza vaccine on the day of discharge;
3) provision of immunization service inside hospital before discharge and a minimum of 1 hour after vaccination and before discharge to observe any potential adverse events of the vaccination.

Comparator
Currently the routine practice is that HF patients discharged from hospitals seek and pay for influenza vaccination service in community health center or immunization clinic at local CDC.

Outcomes
Primary outcome

- Rate of uptake of influenza vaccination among eligible patients in the intervention hospitals and that in the control hospitals;
- Number of patient recruited;
- Rate of adherence to follow-up;

Secondary outcome

- Influenza-like illness (ILI) rate; influenza-like illness is defined as an acute respiratory illness with a measured temperature of ⩾ 38 ℃ and cough, within the past 10 days. [23]
- Death or readmission for heart failure rate over 3 months follow-up through face-to-face or phone call follow-up.

Participant timeline
This pilot study will be conducted between December 2020 and November 2021. Participating centers and participants will be recruited in December 2020. Both hospitals and patients will go through their eligibility screening respectively. Eligible hospitals and patients will then be informed about the study, potential benefits and risks, and be asked for consent. Consented hospitals will be randomized into intervention or control arm before the commencement of the study. Eligible patients will be assessed through collecting their baseline demographics, disease history, and clinical characteristics during hospitalization. On the day of discharge, eligible patients in intervention hospitals will receive influenza vaccine shots. Face-to-face follow-up visits for each patient will be scheduled at 1-month and 3-month post discharge. Study outcome data will be collected during the follow-up visits. If the patient is unable to return for such a visit, they will receive a telephone call instead.

Sample size
As this is a pilot trial and the primary objective is to test the feasibility of increasing influenza vaccination uptake in HF patients, other than to test the efficacy or effectiveness of intervention, a practical number of 11 participating sites and inpatients

with HF during one month (30 patients on average) from each site are set. Intra-cluster correlation coefficient of 3-month death or rehospitalization due to heart failure will be calculated using the outcome of each individual from each cluster. [24]

Recruitment
An estimated average of 30 patients per hospital will be set for recruitment. The admission list from each participating site will be monitored to ensure recruiting consecutive heart failure patients to prevent potential selection bias. If a quota of 50 patients is reached in any given hospital, the recruitment process will end.

Data collection methods
Case report forms (CRFs) at screening, baseline, admission, intervention, vaccination, discharge, 1-month and 3-month follow-up will be designed to collect patient characteristics and study outcomes. Data will be assessed by site investigator team and sampled by steering committee. Outcome data will also be collected for patients who drop out of or drop in to the intervention.

Data Management
An electronic data capture (EDC) system with automatic range checks will be used for data entry and storage. Double entry will be applied to assure data quality.

Randomization
Sequence generation
In this pilot cluster randomized trial, randomization will be applied to participating sites. Computer-generated random numbers for 11 hospitals with 7 in the intervention arm and 4 in the control arm. Site investigators will enroll participants. All eligible patients in intervention hospitals will receive influenza vaccines.

Blinding
This is an open label study.

Analysis
Descriptive analysis will include VCR, number of recruitment, ILI rate and 3-month death or HF readmission in intervention and control hospitals. Clustering effect will be considered when using individual-level data. The proportion of each outcome measure and its confidence interval will be calculated. Analysis will be done at the end of recruitment and the end of follow-up. All statistical analyses will be undertaken using R 3.6.1 (R Foundation for Statistical Computing, Vienna, Austria, https://www.R-project.org/). Schedule of PANDA II pilot is in **Figure 1**.

Data monitoring
We plan to use a combination of central (remote) and site monitoring of the accumulating data. Prior to the initiation of the study at any participating center, all designated research staff will be trained on the study procedures by the operational staff. Data collected by research nurse/coordinator should be monitored and verified every day by operational staff. Mistakes found need to be corrected in time. An

independent Data Safety Monitoring Board (DSMB) will meet every month to review the data quality.

Harms
Every patient in this trail will be insured by a contracted insurer. Adverse Event Following Immunization (AEFI) will be reported to steering committee and the manufacturer pharmacovigilance team. AEFI include vaccine product-related reaction, vaccine quality defect-related reaction, immunization error-related reaction, immunization anxiety-related reaction, and coincidental event. Trial insurer and/or vaccine manufacturer will compensate to those who suffer harm from severe AEFI.

Audit

DSMB will audit the trial conduct during the meeting every six month.

Protocol amendment
We plan to communicate important protocol modifications (e.g. changes to eligibility criteria, outcomes, analyses) to relevant parties (e.g. Investigators, ERC/IRBs, trial participants, trial registries, journals, regulators) on investigators meeting every other week.

Consent
In this pilot cluster randomized trial, we plan to obtain the informed consent from each participating site and each individual participant. Informed consent can only be signed if the patient or designated proxy is fully aware of the risks and possible outcomes.

Confidentiality
Personal information about potential and enrolled participants will be collected, shared and maintained by trial staff in order to protect confidentiality before, during and after the trial. The whole process of data collecting and analyzing will be conducted anonymously.

Declaration of interest
This study will be supported by Sanofi Pasteur.

Access to data
The principal investigator will have full access to study data. A data analytical plan is required if intending to apply for data access.

Dissemination policy
The final conclusion of this pilot trail will be released to public through academic conferences, journals or mass media. The release decision will be made by the study steering committee.

Funding
This study will be supported by Sanofi Pasteur (study code: FLU00144).

Registration
PANDA II pilot will be registered on the Chinese Clinical Trial Registry.

Ethical and regulatory approval
Ethical and necessary regulatory applications will be sent to corresponding institutional review board and/or regulatory authority.

Reference

1. Hao G, Wang X, Chen Z, et al. Prevalence of heart failure and left ventricular dysfunction in China: the China Hypertension Survey, 2012–2015. *European journal of heart failure.* 2019;21(11):1329-1337.
2. Cook C, Cole G, Asaria P, Jabbour R, Francis DP. The annual global economic burden of heart failure. *Int J Cardiol.* 2014;171(3):368-376.
3. Alon D, Stein GY, Korenfeld R, Fuchs S. Predictors and outcomes of infection-related hospital admissions of heart failure patients. *PloS one.* 2013;8(8):e72476-e72476.
4. Drozd M, Garland E, Walker AMN, et al. Infection-Related Hospitalization in Heart Failure With Reduced Ejection Fraction. *Circulation: Heart Failure.* 2020;13(5):e006746.
5. Demicheli V, Jefferson T, Ferroni E, Rivetti A, Di Pietrantonj C. Vaccines for preventing influenza in healthy adults. *The Cochrane database of systematic reviews.* 2018;2:Cd001269.
6. Clar C, Oseni Z, Flowers N, Keshtkar-Jahromi M, Rees K. Influenza vaccines for preventing cardiovascular disease. *The Cochrane database of systematic reviews.* 2015(5):Cd005050.
7. Modin D, Jørgensen ME, Gislason G, et al. Influenza Vaccine in Heart Failure. *Circulation.* 2019;139(5):575-586.
8. Sandoval C, Walter SD, Krueger P, et al. Risk of hospitalization during influenza season among a cohort of patients with congestive heart failure. *Epidemiol Infect.* 2007;135(4):574-582.
9. Ang LW, Yap J, Lee V, et al. Influenza-Associated Hospitalizations for Cardiovascular Diseases in the Tropics. *American Journal of Epidemiology.* 2017;186(2):202-209.
10. Vardeny O, Claggett B, Udell JA, et al. Influenza Vaccination in Patients With Chronic Heart Failure: The PARADIGM-HF Trial. *JACC Heart failure.* 2016;4(2):152-158.
11. Panhwar MS, Kalra A, Gupta T, et al. Effect of Influenza on Outcomes in Patients with Heart Failure. *JACC: Heart Failure.* 2019:979.
12. Bhatt AS, Liang L, DeVore AD, et al. Vaccination Trends in Patients With Heart Failure: Insights From Get With The Guidelines-Heart Failure. *JACC Heart failure.* 2018;6(10):844-855.
13. The macro-epidemiology of influenza vaccination in 56 countries, 1997--2003. *Vaccine.* 2005;23(44):5133-5143.
14. Palache A. Seasonal influenza vaccine provision in 157 countries (2004–2009) and the potential influence of national public health policies. *Vaccine.* 2011;29(51):9459-9466.

15. Hehme N, Colegate T, Palache B, Hessel L. Influenza vaccine supply: building long-term sustainability. *Vaccine.* 2008;26 Suppl 4:D23-26.
16. Yang J, Atkins KE, Feng L, et al. Seasonal influenza vaccination in China: Landscape of diverse regional reimbursement policy, and budget impact analysis. *Vaccine.* 2016;34(47):5724-5735.
17. Yi B, Zhou S, Song Y, et al. Innovations in adult influenza vaccination in China, 2014-2015: Leveraging a chronic disease management system in a community-based intervention. *Human vaccines & immunotherapeutics.* 2018;14(4):947-951.
18. Peng Z, Wang D, Yang J, et al. Current situation and related policies on the implementation and promotion of influenza vaccination in China. *Chinese Journal of Epidemiology.* 2018;39(8):1045-1050.
19. Eldridge SM, Chan CL, Campbell MJ, et al. CONSORT 2010 statement: extension to randomised pilot and feasibility trials. *BMJ.* 2016;355:i5239.
20. Chan A-W, Tetzlaff JM, Altman DG, et al. SPIRIT 2013 statement: defining standard protocol items for clinical trials. *Ann Intern Med.* 2013;158(3):200-207.
21. Chan A-W, Tetzlaff JM, Gøtzsche PC, et al. SPIRIT 2013 explanation and elaboration: guidance for protocols of clinical trials. *BMJ : British Medical Journal.* 2013;346:e7586.
22. Carlson KJ, Lee DCS, Goroll AH, Leahy M, Johnson RA. An analysis of physicians' reasons for prescribing long-term digitalis therapy in outpatients. *Journal of Chronic Diseases.* 1985;38(9):733-739.
23. Fitzner J, Qasmieh S, Mounts AW, et al. Revision of clinical case definitions: influenza-like illness and severe acute respiratory infection. *Bulletin of the World Health Organization.* 2018;96(2):122-128.
24. Rutterford C, Copas A, Eldridge S. Methods for sample size determination in cluster randomized trials. *International journal of epidemiology.* 2015;44(3):1051-1067.

Figure 1. PANDA II pilot trial schedule of enrolment, interventions, and assessments.

	**STUDY PERIOD (November 2020 – April 2021)**					
	**Enrolment**	**Allocation**	**Post-allocation**			**Close-out**
**TIMEPOINT**	***November***	**December**	***January***	***February***	***March***	***April***
**ENROLMENT:**						
**Hospital and patients eligibility screen**	X					
**Hospital and patients informed consent**	X					
**Allocation**		X				
**INTERVENTIONS:**						
[Intervention arm (education + vaccination)]		X				
[Control arm (usual care)]		X				
**ASSESSMENTS:**						
[baseline demographics, clinical characteristics, and vaccination]	X	X				
[recruitment rate]	X	X				
[vaccination logistics, immunization service license]			X	X		
[adherence rate]				X	X	
[influenza-like illness, mortality, readmission]		X	X	X	X	X
