# Supplementary material for: Cluster randomized trial of influenza vaccination in patients with acute heart failure in China: A mixed-methods feasibility study
Source: PLOS Glob Public Health. 2023 Jun 16;3(6):e0001947. doi: 10.1371/journal.pgph.0001947 (PMC10275428; doi:10.1371/journal.pgph.0001947)
Supplement: S1 Text — (DOCX) [file pgph.0001947.s004.docx]

**S1 Text: Intervention details.**

**Intervention**

The intervention will include three components:

1. education to all members of the health care team, physicians, nurses, and patients;
   1. A 30-minute training session to health care teams in all participating hospitals after they are included in the study. Members in a health care team include site investigators, physicians, and nurses. The training session include the association between influenza vaccine and lower risk of mortality and rehospitalization in patients with HF, the study protocol, and current regulations on vaccine circulates. The education material will be designed and delivered by study investigators.
   2. Study nurses in each participating hospital will lead a 15-minute group or individual training session to patients after they reach a relative-stable status during hospitalization. The training sessions include plain language version content on association between influenza infection and cardiovascular disease and current guideline recommendations of influenza vaccine for secondary prevention in patients with established cardiovascular disease. The training material (presentation slides, scripts, and videos) will be designed and delivered by study investigator.

2) provision of free influenza vaccine on the day of discharge.

3) provision of immunization service inside hospital before discharge and a minimum of 1 hour after vaccination and before discharge to observe any potential adverse events of the vaccination.
