# Supplementary material for: Cluster randomized trial of influenza vaccination in patients with acute heart failure in China: A mixed-methods feasibility study
Source: PLOS Glob Public Health. 2023 Jun 16;3(6):e0001947. doi: 10.1371/journal.pgph.0001947 (PMC10275428; doi:10.1371/journal.pgph.0001947)
Supplement: S2 Text — (DOCX) [file pgph.0001947.s005.docx]

**S2 Text: Interview guide for health professionals.**

**RECORD OF INTERVIEW FOR HEALTHCARE PROFESSIONALS.**

**Name of Interviewer:** ____________________________________________

**Name of others present:** ____________________________________________

**Date**: __ __ __ __ / __ __ / __ __

y y y y / m m / d d

**PE ID No:** ____________________________________________

**Role/Job title**: ____________________________________________

**Place of employment: ­­­­­­­____________________________________________**

**Date commenced** __ __ __ __ /__ __

**in current position:** y y y y / m m

**Date graduated** __ __ __ __

y y y y

**Date of birth**: __ __ __ __ / __ __ / __ __

y y y y / m m / d d

**Gender**: Female / Male / Non Binary

**RESEARCHER FIELD NOTES:**

How do you think the interview went?

What struck you as important?

What further questions/areas would you like to explore in the next interview?

**INTERVIEW GUIDE FOR HEALTHCARE PROFESSIONALS (PHYSICIANS, NURSES, HOSPITAL MANAGERS, CENTER FOR DISEASE CONTROL STAFFS, IMMUNIZATION PRGRAM PLANNER, INSURER)**

**Overall aim of the process evaluation interviews:** To understand for whom, what worked, what didn’t work, why and why not in preventing influenza infection, and improving health outcomes for heart failure patients, and to inform future implementation.

**Say to the healthcare professional**:

*Hi, I am [name] and I’m from [organisation]. Thank you for taking part in this interview. As discussed, we are trying to find out how we can better care for people with heart disease. You can change your mind about talking to me at any time before or during the interview and stop the interview at any time. Are you happy to continue? [If no, thank them for their time and end interview; if yes continue.] Thank you [name] for agreeing to take part.*

*We are interviewing health professionals involved in the care of heart failure patients. We will use your feedback and the feedback of others to write a summary of what people have told us. We ask that you do not name any patients in our interview when discussing the care you provide. Are you happy for me to record the interview? I will keep the file in a secure location until we finish writing the report and then it will be destroyed. Our interview will be transcribed professionally and we will ensure your privacy and confidentiality.*

*Do you have any questions before we start?*

*Note: Key questions in bold, with probing questions in non-bold. Questions do not have to be asked in this order, and not all questions have to be covered.

| **Domain 1: To understand the context of the site and health system, and how preventing influenza infection for heart failure patients fits within this** |
| --- |
| **Warm up: What is your role? Can you describe your role in the PANDA II Pilot Study? How did you get involved and why? [aim for a longish narrative]**  **What do you think are the main purposes of the PANDA II Pilot Study?** [Probe: increase access and coverage of influenza vaccine, infection prevention, bridging preventive care and therapeutic treatment, and coordination of duties from different sectors in a health system]   - What types of patients would need the free in-hospital influenza vaccine? Why is that? [probe: patient level factors such as access to immunization clinics, insurance type, health literacy, socio-demographics] - How much responsibility do you think should be on the individual patient for managing their own health? - Do you think the patients like the free in-hospital influenza vaccine? Why or why not? - Do you like the education session? Why or why not? How much did you use it?   **What is the usual in-hospital care for patients with heart failure from your hospital?** [probe: education re: infection prevention, about access to influenza vaccine]  **What do you think are the main causes of death or hospital readmissions within 1 year for heart failure patients? Are they preventable in any way?** [probe: patient level factors such as lack of knowledge, inconvenient access to preventive care; health care level factors such as neglected preventive care, access to preventive care for hospitalized patients] |
| **Domain 2: To understand the implementation barriers and facilitators** |
| **What in your perspective has gone well in the implementation of the trial? What in your perspective has been difficult? Why is that? Can you provide some examples?**   - Do you think you have kept to the protocol of the trial in terms of the study design and processes (fidelity)? Have you adapted the implementation in anyway? - Did you recruit patients that you wanted for the trial? How was that process?   **What do you think of the adoption of the its use from the patients’ and health providers’ perspective?**   - Is it adequate or not, and why? Can it be improved? - Was there an opportunity to improve it’s implementation? For e.g. the adoption of the in-hospital Point of Vaccination (PoV) and free influenza vaccine policy   **Were there any health system issues that impacted upon the implementation? [**probe about: workforce, health financing, governance, fitting in with current information technology such as eMR] |
| **Domain 3: To understand the outcomes and future implementation** |
| **Do you think this trial will be successful? Why is that?**   - Would it improve influenza vaccine coverage rate? - Would it improve patient awareness of influenza infection? Why is that? - Upon hindsight, is there anything that you would do differently?   **Would it be beneficial and feasible to continue and embed free in-hospital influenza vaccine into usual care?**   - Do you think this will be widely accepted by patients and health providers in a public hospital? Why or why not? - How do you think heart failure patients could be best supported during hospitalization? Do you see free in-hospital influenza vaccine playing a part in this process? |
| **Concluding question and Statement** |
| Is there anything else you would like to say that we have not talked about in this interview?  Thank you so much for your time and for sharing your insights. |
