# Supplementary material for: Cluster randomized trial of influenza vaccination in patients with acute heart failure in China: A mixed-methods feasibility study
Source: PLOS Glob Public Health. 2023 Jun 16;3(6):e0001947. doi: 10.1371/journal.pgph.0001947 (PMC10275428; doi:10.1371/journal.pgph.0001947)
Supplement: S3 Text — (DOCX) [file pgph.0001947.s006.docx]

**S3 Text: Interview guide for patients.**

**RECORD OF INTERVIEW FOR TRIAL PARTICIPANTS.**

**Name of Interviewer:** ____________________________________________

**Name of others present:** ____________________________________________

**Date**: __ __ __ __ / __ __ / __ __

y y y y / m m / d d

**PE ID No:** ____________________________________________

**Date of birth**: __ __ __ __ / __ __ / __ __

y y y y / m m / d d

**Hospital**: ____________________________________________

**GROUP ALLOCATED: Intervention  Usual care**

**Patient vaccination status: Vaccinated  Not-vaccinated**

**Gender**: Female / Male / Non Binary

**RESEARCHER FIELD NOTES:**

How do you think the interview went?

What struck you as important?

What further questions/areas would you like to explore in the next interview?

**INTERVIEW GUIDE FOR PARTICIPANTS**

**Overall aim of the process evaluation interviews:** To understand for whom, what worked, what didn’t work, why and why not in preventing influenza infection, and improving health outcomes for heart failure patients, and to inform future implementation.

**Say to the participant**:

*Hi, I am [name] and I’m from [organisation]. Thank you for taking part in this interview. As discussed, we would like to ask you questions about your care from hospital and also in relation to infection prevention. You can change your mind about talking to me at any time before or during the interview and stop the interview at any time. Are you happy to continue? [If no, thank them for their time and end interview; if yes continue.] Thank you [name] for agreeing to take part.*

*We will use your feedback and the feedback of others to write a summary of what people have told us. There will be absolutely no identification of any real names or identification of where you live or which health professionals you have seen. Are you happy for me to record the interview? I will keep the file in a secure location until we finish writing the report and then it will be destroyed. Our interview will be transcribed professionally and we will ensure your privacy and confidentiality.*

*Do you have any questions before we start?*

*Note: Key questions in bold, with probing questions in non-bold. Questions do not have to be asked in this order, and not all questions have to be covered.

| **Domain 1: To understand the patient context and usual care** |
| --- |
| **Can you tell us about your recent admission into the hospital for heart failure?**   - When were you diagnosed with heart failure? How has that been managed? - What treatment did you receive?   **When you were discharged, what were some key things that you were advised to do? How was it to follow this advice? What went well or not?**   - Preventing infection? What do you think about influenza vaccine? How useful is it and how easy is it to get? - What do you think about the cost of preventive care? |
| **Domain 2: Intervention implementation and mechanisms**  **ONLY FOR Intervention Group: *As you know, we are exploring if the intervention that you received were helpful. The next questions will explore this care you received in greater detail. Please feel free to be honest about what it was like for you, as any feedback you provide will help us improve the care we provide.*** |
| **Can you tell me about the intervention that you received?**  **What did you like about it?**   - Did it help you to understand the association between influenza infection and your heart failure, and how you could manage it? Can you provide an example of how that happened? - How did you feel about the free in-hospital influenza vaccine service?   **What didn’t you like?**   - Could you provide an example of what happened?   **Has your symptoms or condition improved or deteriorated?**   - Were these changes in your condition due to the intervention? - Would you recommend influenza vaccine for other patients with heart failure?   **What else would you liked to have received as part of the intervention?**   - Are there any therapies you didn’t receive that you wanted to try? (e.g. pneumonia vaccine, or education on preventive care etc) |
| **Domain 3: Exploring more about mechanisms of the patients’ outcomes** |
| **Do you think influenza vaccine will help improve your health?**   - Given the current pandemic, will infection prevention become more a priority for you? - Will you look for it next year? - What did you like/dislike about follow up? - Can your health providers do more for you? In what way?   **How are you managing now?**   - Have you been readmitted to hospital since you first enrolled in the study? Why was that? - How are you managing to keep well, and manage your daily activities? (probe: self-care, and other supports) - What has been difficult? What has been going well? |
| **Concluding question and Statement** |
| Is there anything else you would like to say that we have not talked about in this interview?  Thank you so much for your time and for sharing your insights. |
