# Supplementary material for: Cluster randomized trial of influenza vaccination in patients with acute heart failure in China: A mixed-methods feasibility study
Source: PLOS Glob Public Health. 2023 Jun 16;3(6):e0001947. doi: 10.1371/journal.pgph.0001947 (PMC10275428; doi:10.1371/journal.pgph.0001947)
Supplement: S1 Table — (DOCX) [file pgph.0001947.s007.docx]

**S1 Table 4: Regression models output summary for outcomes.**

|  | Intervention versus Control | | |
| --- | --- | --- | --- |
| Outcomes | Unadjusted OR (95% CI) | Covariates | Adjusted OR (95% CI) |
| Influenza vaccination | 1991.2 (320.0, 41555.0) | Intervention | 2516.3 (229.1, 27634.0) |
|  |  | Age | 0.99 (0.95, 1.03) |
|  |  | Male | 0.78 (0.33, 1.86) |
|  |  | Primary school or lower education | 0.95 (0.35, 2.57) |
|  |  | Ejection fraction $\geq$ 50% | 1.72 (0.61, 4.87) |
|  |  | CHD | 2.12 (0.87, 5.13) |
|  |  | Hypertension | 1.25 (0.56, 2.80) |
|  |  | Diabetes | 0.56 (0.22, 1.42) |
|  | Intervention versus Control | | |
| Outcomes | Unadjusted HR (95% CI) | Covariates | Adjusted HR (95% CI) |
| Time to 3-month follow-up death or HF readmission (day) | 0.94 (0.57, 1.55) | Intervention | 0.92 (0.55, 1.55) |
|  |  | Age | 1.01(1.00, 1.02) |
|  |  | Male | 1.05 (0.79, 1.39) |
|  |  | Primary school or lower education | 0.69 (0.50, 0.94) |
|  |  | Ejection fraction $\geq$ 50% | 0.64 (0.35, 1.18) |
|  |  | CHD | 1.70 (1.24, 2.33) |
|  |  | Hypertension | 0.73 (0.55, 0.97) |
|  |  | Diabetes | 0.79 (0.48, 1.28) |
