# Supplementary material for: Cluster randomized trial of influenza vaccination in patients with acute heart failure in China: A mixed-methods feasibility study
Source: PLOS Glob Public Health. 2023 Jun 16;3(6):e0001947. doi: 10.1371/journal.pgph.0001947 (PMC10275428; doi:10.1371/journal.pgph.0001947)
Supplement: S2 Table — (DOCX) [file pgph.0001947.s008.docx]

**S2 Table 5: Interviewees characteristics.**

|  | Types of interviewees | | |
| --- | --- | --- | --- |
| Variable | All (N = 51) | Patient (n1 = 13) | Health professional (n2 = 38) |
| From intervention site | 28 (55%) | 7 (53.8%) | 21 (55.3%) |
| Age | 47 (41, 57) | 71 (65, 79) | 45(38, 50) |
| Female | 19 (37%) | 5 (38.5%) | 14 (36.8%) |
| Face-to-face interviews | 47 (92%) | 10 (76.9%) | 37 (97.4%) |
| Hospital | - | - | 24 (47%) |
| Physicians | - | - | 6 |
| Nurses | - | - | 5 |
| Hospital dean or department head | - | - | 13 |
| Patients | - | 13 (25%) |  |
| CDC or health commission | - | - | 9 (18%) |
| Insurance bureau | - | - | 5 (10%) |
| Length of years having been on the current position | - | - | 7 (4) |
| Vaccinated patient in the control site | - | 1 | - |
| Unvaccinated patient in the intervention site | - | 1 | - |

CDC, center for disease control and prevention.
