# Supplementary material for: Cluster randomized trial of influenza vaccination in patients with acute heart failure in China: A mixed-methods feasibility study
Source: PLOS Glob Public Health. 2023 Jun 16;3(6):e0001947. doi: 10.1371/journal.pgph.0001947 (PMC10275428; doi:10.1371/journal.pgph.0001947)
Supplement: S3 Table — (DOCX) [file pgph.0001947.s009.docx]

**S3 Table 6:** **Additional illustrative quotes from interviewees.**

| Interviewee Number | Occupation | Code | Quote | |
| --- | --- | --- | --- | --- |
| 16 | Doctor from intervention hospital | Lack of education | Being a clinical doctor and treating patients every day, I sometimes found myself lacking the knowledge of disease prevention and control. I had no idea that there were vaccines until I heard about it from CDC staffs and education workshops. | |
| 27 | Doctor from intervention hospital |  | He/she (the patient) sometimes comes to the hospital with heart failure and shortness of breath. He/she believes he/she had heart failure rather than a cold. He/she is unaware of the importance of vaccination. | |
| 15 | Public health professional from control county |  | Many doctors do not know about vaccines. To be honest, their knowledge level was not high. These doctors got no idea about what the vaccine is, therefore, they did not recommend the vaccine to their patients. If they recommend the vaccine, many patients would be likely to take it. | |
| 2 | Nurse from intervention hospital |  | Then, through this program, explain to the patient. He/she (patient) would understand after the explanation that the vaccine could not be taken whenever you wished (supply constraints and during flu season). The patient showed that he/she was aware of the situation and that he/she was pleased to be vaccinated. | |
| 51 | Health commission staff from control county | Vaccine cost | For most people, a hundred RMB is a large cost. They have a lot to bear. If those in authority could provide financial assistance or medical insurance support, the work would be easily achieved. | |
| 29 | Nurse from intervention hospital | Vaccine safety | Most people would like to be vaccinated. Some individuals were hesitant to get the vaccine because they were concerned about side effects. Even with further information, still a minority of patients refused to be vaccinated and among them older adults were more prevalent. | |
| 26 | Patient from intervention hospital | Vaccine efficacy | I think it is useless to get influenza vaccine. I don’t always get cold during winter, so it is the same for me no matter I get influenza vaccination or not. |  |
| 18 | Doctor from intervention hospital |  | One of the patient’s relatives works in the health care system and did not want the patient to take the vaccine. | |
| 31 | Public health professional from control county |  | After the Changsheng vaccine event in 2018 (news on side effect cases from a vaccine manufacturer in China) and the Shandong medical misconduct (medical incident happened in a hospital), many medical personnel became resistant to immunization services. | |
| 18 | Doctor from intervention hospital | Organizational coordination | Our cardiology department chair and the head of the hospital immunization service department consult with each other in terms of vaccination for patients. | |
| 49 | Public health staff from control county |  | To establish a new temporary vaccination facility, we will need to have 1) refrigeration, 2) observation space after vaccination, and 3) staff assigned and trained for vaccination in place and to pass the inspection from regulatory authority. They will give temporary vaccination credentials to us. | |
| 19 | Nurse from intervention hospital |  | They (doctors and nurses on clinical roster) are really busy and have no spare time. It will be 11 a.m. when they complete handover to the next shift to give vaccination. They (patients) won’t be able to receive the vaccine due to a lack of time.”  “It needs to be managed by a designated individual. This individual could gain a better understanding of the situation so that he or she could assume responsibility for every patient.” | |
| 28 | Health insurance bureau officer in intervention county | COVID-19 | Seniors in my family had less cold during COVID-19, because they seldomly went out. If they had to go out, they wore masks, so they were pretty good throughout last year (2020). | |
| 15 | Public health professional from control county |  | Because of COVID-19, people are eager to be vaccinated. We ordered the vaccine earlier this year than in previous years. | |
| 21 | Public health profession from intervention county |  | It was a temporary PoV for COVID-19 vaccine at this hospital. Because their (site hospital leadership) request for influenza vaccination, we administratively added influenza vaccination into their PoV function. | |
| 19 | Nurse from intervention hospital |  | I was in cardiac rehabilitation unit before. Due to the pandemic, the number of patients for rehabilitation was low in this year. Therefore, my task was shifted to influenza vaccination for this study. | |
| 1 | Hospital manager from intervention site | Integration of healthcare and preventive services | As a result of national fundamental policies, (the hospital) now prioritize prevention and combines prevention with treatment. This is a great project that we can easily embrace. | |
| 4 | Doctor from intervention hospital |  | After patients in our unit were vaccinated and started to do this program, we know that people could benefit from the vaccine. | |
